# Global stability analysis of an SVEIR epidemic model with general incidence rate

**DOI:** 10.1186/s13661-018-0961-7

**Published:** 2018-03-27

**Authors:** Da-peng Gao, Nan-jing Huang, Shin Min Kang, Cong Zhang

**Affiliations:** 1 School of Mathematics and Information, China West Normal University, Nanchong, P.R. China; 2 Department of Mathematics, Sichuan University, Chengdu, P.R. China; 3 Department of Mathematics and the RINS, Gyeongsang National University, Jinju, Korea; 4 School of Management, Sichuan University of Science and Engineering, Zigong, P.R. China

**Keywords:** 92D25, 92D30, 34D23, 37B25, Epidemic model, Reproduction number, Lyapunov function, Geometric approach, Global stability, Susceptible–Vaccinated–Exposed–Infectious–Recovered

## Abstract

In this paper, a susceptible-vaccinated-exposed-infectious-recovered (SVEIR) epidemic model for an infectious disease that spreads in the host population through horizontal transmission is investigated, assuming that the horizontal transmission is governed by an unspecified function $f(S,I)$. The role that temporary immunity (vaccinated-induced) and treatment of infected people play in the spread of disease, is incorporated in the model. The basic reproduction number $\mathcal{R}_{0}$ is found, under certain conditions on the incidence rate and treatment function. It is shown that the model exhibits two equilibria, namely, the disease-free equilibrium and the endemic equilibrium. By constructing a suitable Lyapunov function, it is observed that the global asymptotic stability of the disease-free equilibrium depends on $\mathcal{R}_{0}$ as well as on the treatment rate. If $\mathcal{R}_{0}>1$, then the endemic equilibrium is globally asymptotically stable with the help of the Li and Muldowney geometric approach applied to four dimensional systems. Numerical simulations are also presented to illustrate our main results.

## Introduction

Mathematical modeling enjoys popularity in both preventing and controlling infectious diseases such as severe acute respiratory syndrome (SARS) [1], human immunodeficiency virus infection/acquired immune deficiency syndrome (HIV/AIDS) [2], H5N1 (avian flu) [3] and H1N1 (swine flu)[4]. In recent years, a lot of efforts have been made to develop realistic diseases and further study the asymptotic behavior of such epidemic models [5]. In the field of studying epidemic model behavior, one of the most important parts is to analyze steady states together with their stability [6]. In general, there are two distinct techniques named Lyapunov’s direct method and Li–Muldowney’s geometric approach to give sufficient conditions of global stability for the equilibrium states (see, for example, [7–14]). We would like to mention some related work concerned with the existence of positive solutions for the discrete fractional boundary value problem [15], the sensitivity analysis for optimal control problems governed by nonlinear evolution inclusions [16] and the nonexistence of global in time solution of the mixed problem for the nonlinear evolution equation with memory generalizing the Voigt–Kelvin rheological model [17].

It is well known that the rate of incidence plays the main part in modeling infectious diseases. The rise and fall of epidemics can be influenced by some factors, such as density of population and life style [18, 19]. Many researchers have adopted different nonlinear incidence rates in their works. For more details, we refer the reader to [8–14, 20–34] and the references therein. When it comes to control of a disease, it is generally known that the spread of many diseases can be prevented by vaccinating. When massive vaccination is impossible, the second stage of defensive mechanism could be medical treatment. Individuals need to bear in mind that the treatment is an indispensable way to take precautions for some diseases (for instance, measles, phthisis and influenza). In recent years, many treatment functions have been introduced by several authors to study some epidemic models under different conditions (see, for instance, [12, 14, 27, 31, 35–38]).

Recently, Dénes and Röst [27] investigated the following *SI* model:

1.1 $$ \textstyle\begin{cases} \frac{dS}{dt} =\mu-f(S,I)-\mu S, \\ \frac{dI}{dt} =f(S,I)-g(I), \\ 1 =S+I+R, \end{cases} $$

where a population of constant size (assumed to be equal to 1) is divided into three compartments: susceptible (denoted by *S*), infected (denoted by *I*) and recovered (denoted by *R*). The transmission of the infection is governed by the incidence rate $f(S,I)$ and *μ* is birth rate as well as the death rate of the susceptible class. The nonlinear function $g(I)$ denotes the sum of the death rate and the recovery rate for the infected individuals satisfying $g(0)=0$ and $g(I)>0$ for $I>0$. Using a Dulac function approach, which aims at eliminating the existence of the periodic solution and proving the global stability by the Poincaré-Bendixson theorem (see [39], p. 54), they obtained the global stability of the disease-free equilibrium and the endemic equilibrium for system (1.1).

Very recently, Upadhyay et al.[12] considered the following e-epidemic model:

1.2 $$ \textstyle\begin{cases} \frac{dS}{dt} =A-\delta_{0} S-\frac{\alpha SI}{S+I+c}+\eta V-\mu S, \\ \frac{dE}{dt} =\frac{\alpha SI}{S+I+c}-(\delta_{0}+\delta_{1})E, \\ \frac{dI}{dt} =\delta_{1}E-(\delta_{0}+\delta_{2}+\delta_{3})I-\frac{\beta I}{I+a}, \\ \frac{dR}{dt} =\delta_{2}I-\delta_{0}R+\frac{\beta I}{I+a}, \\ \frac{dV}{dt} =\mu S-(\delta_{0}+\eta)V. \end{cases} $$

with initial conditions: $S(0)=S_{0}>0$, $E(0)=E_{0}\geq0$, $I(0)=I_{0}\geq0$, $R(0)=R_{0}\geq0$ and $V(0)=V_{0}\geq0$. All the parameters in model (1.2) are positive and are defined as follows: *S*, *E*, *I*, *R* and *V* represent the number of susceptible nodes, exposed nodes, infectious nodes, recovered nodes and vaccinated nodes at time *t*, respectively; *A* is the recruitment rate of new nodes, *c* is the half saturation constant for susceptible nodes *S*, *α* is the contact rate or the rate of transfer of virus from an infectious node to the susceptible node, *η* is rate at which the vaccinated nodes lose their immunity and join the susceptible class, *β* is the maximal treatment capacity of a network, $\delta_{0}$ is the natural crashing rate of nodes all classes, *a* is the half saturation constant for an infected node *I*, *μ* is the vaccination rate coefficient, $\delta_{3}$ is the virus induced crashing rate and $\delta_{1}$, $\delta_{2}$ are the state transition rates. Using a Lyapunov function and a geometric approach, they obtained the global stability of virus-free equilibrium and endemic equilibrium for system (1.2).

As pointed out by Liu and Yang [11], due to the high similarity between computer virus and biological virus, it is acceptable to establish dynamical models describing biological virus among a population by modifying an e-epidemic model. Thus, it is interesting and important to extend model (1.2) to study the biological virus in the infectious disease.

Inspired by these research results above, in this paper, we consider the following system with five compartments:

1.3 $$ \textstyle\begin{cases} \frac{dS}{dt} =A-\delta_{0} S-f(S,I)+\eta V-\mu S, \\ \frac{dE}{dt} =f(S,I)-(\delta_{0}+\delta_{1})E, \\ \frac{dI}{dt} =\delta_{1}E-(\delta_{0}+\delta_{2}+\delta_{3})I-g(I), \\ \frac{dR}{dt} =\delta_{2}I-\delta_{0}R+g(I), \\ \frac{dV}{dt} =\mu S-(\delta_{0}+\eta)V, \end{cases} $$

where $S(t)$, $E(t)$, $I(t)$, $R(t)$, $V(t)$ are the number of susceptible population, exposed population, infective population, recovered population, vaccinated population, respectively. The two-variable function $f(S,I)$ represents incidence rate and the nonlinear function $g(I)$ denotes the removal rate of infective individuals because of the treatment of infective. The initial conditions for system (1.3) are as follows:

1.4 $$ \begin{aligned} &S(0)=S_{0}\geq0,\qquad E(0)=E_{0}\geq0,\qquad I(0)=I_{0}\geq0, \\ &R(0)=R_{0}\geq0,\qquad V(0)=V_{0}\geq0. \end{aligned} $$

Clearly, $N=S(t)+E(t)+I(t)+R(t)+V(t)$ denotes the total number of high-risk human population at time *t*.

The model parameters of system (1.3) are described as follows:

*A*: the rate at which new individuals (including newborns and immigrants) enter the susceptible population,
$\delta_{0}$:
natural death rate of population all classes,
*η*: the rate at which the vaccinated population lose their immunity and join the susceptible class,
*μ*: vaccination rate coefficient,
$\delta_{1}$:
the rate at which exposed population become infective,
$\delta_{2}$:
natural recovery rate of infective population,
$\delta_{3}$:
disease-related death rate of infective population.

Model (1.3) involves certain assumptions which consist of the following:

(i) The new individuals enter the population with a constant rate and all the new individuals are susceptible.
(ii) Susceptible individuals move to exposed class by adequate contact with infective individuals and after some time (i.e., latency period), they become infectious and move to infectious class.
(iii) The infectious individuals are assumed to leave the infectious class as a result of natural death and disease-related death as well as recovery of infected individuals.
(iv) After recovery the individuals become immunized and hence they are no longer susceptible to it.
(v) It is assumed that a fraction of susceptible individuals get vaccinated and join the vaccinated class. A part of vaccinated individuals may lose their immunity and rejoin the susceptible class.

It is easy to see that system (1.3) includes (1.1) and (1.2) as special cases and so model (1.3) provides a uniform setting for the computer virus and biological virus studies. Following the classical assumptions [27, 40], it is reasonable to suppose that the transmission of the infection is governed by an incidence rate $f(S,I)$ in model (1.3). Moreover, as pointed out by Wang [31], the recovery rate is naturally dependent on the number of infected individuals provided the health care resources are constrained and so it is natural to use the nonlinear function $g(I)$ as the treatment function in model (1.3).

The main purpose of this paper is to derive the expression for the basic reproduction number and further show the global stability of disease-free as well as endemic equilibria by the aid of Lyapunov function and Li–Muldowney geometric approach applied to four dimensional systems. This paper is organized as follows. In Sect. 2, some elementary assumptions on the functions *f* and *g* will be given, and the basic reproduction number $R_{0}$ is provided. Also the equilibrium points are discussed. The global stability of disease-free equilibrium and endemic equilibrium are analyzed in Sects. 3 and 4, respectively. All our important analytical findings are numerically verified with the help of Mathlab in Sect. 5. Finally, a brief conclusion is given in Sect. 6.

## Basic reproduction number and equilibrium

To define the basic reproduction number $\mathcal{R}_{0}$ and indicate the existence of equilibrium, we give some hypotheses.

1. $f:\mathbb{R}_{+}^{2}\rightarrow\mathbb{R}_{+}$ is differentiable such that
  $f(S,0)=f(0,I)=0$ for all $S,I\geq0$;
  $f(S,I)>0$ for all $S,I>0$;
  $\frac{\partial f(S,I)}{\partial S}>0$ for all $S\geq0$ and $I>0$;
  $\frac{\partial f(S,I)}{\partial I}\geq0$ for all $S,I\geq0$;
  $I\frac{\partial f(S,I)}{\partial I}-f(S,I)\leq0$ for all $S,I\geq0$.
2. $g:\mathbb{R}_{+}\rightarrow\mathbb{R}_{+}$ is differentiable such that $g(0)=0$, $g'(I)>0$ and $g''(I)\leq0$ for $I\geq0$.
3. $\frac{d}{dI}(\log\frac{g(I)}{f_{S}(I)})\geq0$ holds for all $S,I>0$, where $f_{S}(I):=f(S,I)$ for $S,I>0$.

### Remark 2.1

1. It is easy to check that the classes of $f(S,I)$ satisfying (H1) include incidence rates such as
  $$f(S,I)=\frac{\beta SI}{1+aI^{q}}, \qquad f(S,I)=\frac{\beta SI}{1+aS+bI},\qquad f(S,I)= \frac{\beta SI}{1+aS+bI+abSI}, $$
  for $\beta,a,b>0$ and $0\leq q\leq1$.
2. It is straightforward to show that the classes of $g(I)$ satisfying (H2) include removal rates such as
  $$g(I)=\frac{rI}{1+bI}, \qquad g(I)=\frac{rI}{I+a},\qquad g(I)=r\arctan I, $$
  for $r>0$, $a>0$, $b>0$.
3. By hypothesis (H2), we know that $\varPhi (I)=\frac{g(I)}{I}$ is a monotone decreasing function on $I>0$.
4. The assumption (H3) is equivalent to the following inequality:
  $$\frac{\partial}{\partial I}f(S,I)g(I)\leq f(S,I)\frac{d}{dI}g(I), $$
  which can be found in [27].
5. By the assumptions, it is easy to find that system (1.3) always has a disease-free equilibrium point $P_{0}=(S_{0},0,0,0,V_{0})$, where
  $$S_{0}=\frac{(\delta_{0}+\eta)A}{\delta_{0}^{2}+(\mu+\eta)\delta_{0}},\qquad V_{0}=\frac {A\mu}{\delta_{0}^{2}+(\mu+\eta)\delta_{0}}. $$

We shall assume that (H1), (H2) and (H3) hold in the rest of this paper.

Now we define the basic reproduction number $\mathcal{R}_{0}$ for model (1.3) as follows:

$$\mathcal{R}_{0}=\frac{\delta_{1}}{m_{2}(m_{3}+g'(0))}\frac{\partial f}{\partial I} \biggl( \frac{A}{m_{1}-\frac{\mu\eta}{m_{4}}},0 \biggr), $$

where

$$m_{1}=\delta_{0}+\mu,\qquad m_{2}= \delta_{0}+\delta_{1},\qquad m_{3}= \delta_{0}+\delta_{2}+\delta _{3},\qquad m_{4}=\delta_{0}+\eta. $$

In order to find the positive equilibria of model (1.3), set

2.1 $$ \textstyle\begin{cases} A-f(S,I)-m_{1}S+\eta V =0, \\ f(S,I)-m_{2}E =0, \\ \delta_{1}E-m_{3}I-g(I) =0, \\ \delta_{2}I-\delta_{0}R+g(I) =0,\qquad \mu S-m_{4}V =0. \end{cases} $$

It follows that $A-m_{1}S+\eta V=m_{2}E=m_{2}\frac{m_{3}I+g(I)}{\delta_{1}}$ and $V=\frac{\mu S}{m_{4}}$.

Substituting the above equalities into the second equation in (2.1), one has

$$f \biggl(\frac{A-\frac{m_{2}m_{3}I+m_{2}g(I)}{\delta_{1}}}{m_{1}-\frac{\mu\eta }{m_{4}}},I \biggr)=m_{2}\frac{m_{3}I+g(I)}{\delta_{1}}. $$

Let

2.2 $$ F(I)=f \biggl(\frac{A-\frac{m_{2}m_{3}I+m_{2}g(I)}{\delta_{1}}}{m_{1}-\frac{\mu\eta }{m_{4}}},I \biggr)-m_{2} \frac{m_{3}I+g(I)}{\delta_{1}}. $$

Then it is easy to see that the positive equilibrium points of system (2.1) are given by zeros of *F* in the interval $(0,\frac{A\delta _{1}}{m_{3}m_{2}}]$.

We denote $G(I)=m_{3}I+g(I)-\frac{A\delta_{1}}{m_{2}}$ for convenience. Then it is easy to see

$$G(0)=-\frac{A\delta_{1}}{m_{2}}< 0,\qquad G\biggl(\frac{A\delta_{1}}{m_{3}m_{2}}\biggr)=g\biggl( \frac {A\delta_{1}}{m_{3}m_{2}}\biggr)>0. $$

Therefore, we conclude that $G(I)$ has at least one root named *Ĩ* in the interval $(0,\frac{A\delta_{1}}{m_{3}m_{2}}]$. That is, $m_{3}\tilde {I}+g(\tilde{I})=\frac{A\delta_{1}}{m_{2}}$ and so

$$ F(\tilde{I})=f \biggl(\frac{A-\frac{m_{2}m_{3}\tilde{I}+m_{2}g(\tilde {I})}{\delta_{1}}}{m_{1}-\frac{\mu\eta}{m_{4}}},\tilde{I} \biggr)-m_{2} \frac {m_{3}\tilde{I}+g(\tilde{I})}{\delta_{1}} =-\frac{m_{2}\frac{A\delta_{1}}{m_{2}}}{\delta_{1}} =-A< 0. $$

Since $f(S,0)=0$, we know that $\frac{\partial f}{\partial S} (\frac {A}{m_{1}-\frac{\mu\eta}{m_{4}}},0 )=0$ and so

$$ F'(0)=\frac{\partial f}{\partial I} \biggl(\frac{A}{m_{1}-\frac{\mu\eta }{m_{4}}},0 \biggr)- \frac{m_{2}}{\delta_{1}}\bigl(m_{3}+g'(0)\bigr)= \frac{m_{2}}{\delta _{1}} \bigl(m_{3}+g'(0) \bigr) ( \mathcal{R}_{0}-1). $$

If $\mathcal{R}_{0}>1$, then system (2.1) has a positive equilibrium point $P^{*}=(S^{*},E^{*},I^{*},R^{*},V^{*})$, where

$$\begin{aligned}& S^{*}=\frac{A-\frac{m_{2}m_{3}I^{*}+m_{2}g(I^{*})}{\delta_{1}}}{m_{1}-\frac{\mu\eta }{m_{4}}},\qquad E^{*}=\frac{m_{3}I^{*}+g(I^{*})}{\delta_{1}}, \\& R^{*}=\frac{\delta_{2}I^{*}+g(I^{*})}{\delta_{0}},\qquad V^{*}=\frac{\mu S^{*}}{m_{4}}. \end{aligned}$$

In the following, we show that $P^{*}$ is the unique positive equilibrium point of system (2.1). For any positive equilibrium point $P^{*}$, by (2.2) and hypotheses (H1) and (H2), we have

2.3 $$\begin{aligned} F'\bigl(I^{*}\bigr) =&\frac{\partial f(S^{*},I^{*})}{\partial S}\frac {(-m_{2})(m_{3}+g'(I^{*}))}{\delta_{1}(m_{1}-\frac{\mu\eta}{m_{4}})}+ \frac{\partial f(S^{*},I^{*})}{\partial I} \\ &{}-\frac{m_{2}(m_{3}+g'(I^{*}))}{\delta_{1}}. \end{aligned}$$

Since $m_{1}m_{4}=(\delta_{0}+\mu)(\delta_{0}+\eta)>\mu\eta$ and hypotheses (H1) and (H2) hold, we have

2.4 $$ \frac{\partial f(S^{*},I^{*})}{\partial S}\frac{1}{m_{1}-\frac{\mu\eta }{m_{4}}} \biggl(-\frac{1}{\delta_{1}} \biggr) \bigl(m_{2}m_{3}+m_{2}g' \bigl(I^{*}\bigr) \bigr)< 0. $$

Since $g(0)=0$ and *g* is differentiable on $\mathbb{R}_{+}$, there exists $\xi\in(0,I^{*})$ such that $\frac{g(I^{*})}{I^{*}}=g^{\prime}(\xi)$. By using hypotheses (H2) and (H3), one has

2.5 $$\begin{aligned} \frac{\partial f(S^{*},I^{*})}{\partial I}-\frac{m_{2}}{\delta_{1}}\bigl(m_{3}+g' \bigl(I^{*}\bigr)\bigr) =&\frac{\partial f(S^{*},I^{*})}{\partial I}-\frac {f(S^{*},I^{*})}{m_{3}I^{*}+g(I^{*})} \bigl(m_{3}+g'\bigl(I^{*}\bigr)\bigr) \\ < &\frac{f(S^{*},I^{*})g'(I^{*})}{g(I^{*})}-\frac {f(S^{*},I^{*})(m_{3}+g'(I^{*}))}{m_{3}I^{*}+g(I^{*})} \\ =&f\bigl(S^{*},I^{*}\bigr) \biggl[\frac{g'(I^{*})}{g(I^{*})}-\frac {m_{3}+g'(I^{*})}{m_{3}I^{*}+g(I^{*})} \biggr] \\ =&f\bigl(S^{*},I^{*}\bigr)\frac{m_{3}I^{*}g'(I^{*})-m_{3} g(I^{*})}{g(I^{*})[m_{3}I^{*}+g(I^{*})]} \\ =&f\bigl(S^{*},I^{*}\bigr)\frac{m_{3}I^{*}[g'(I^{*})-\frac {g(I^{*})}{I^{*}}]}{g(I^{*})[m_{3}I^{*}+g(I^{*})]} \\ =&f\bigl(S^{*},I^{*}\bigr)\frac{m_{3}I^{*}[g'(I^{*})-g'(\xi )]}{g(I^{*})[m_{3}I^{*}+g(I^{*})]} \\ < & 0. \end{aligned}$$

Thus, it follows from (2.3), (2.4) and (2.5) that $F'(I^{*})<0$.

Suppose that there exists another positive equilibrium point $P_{1}=(S_{1},E_{1},I_{1},R_{1},V_{1})$. Then $F'(I_{1})\geq0$ due to the property of continuous function. This is a contradiction. Therefore, system (2.1) has a unique endemic equilibrium $P^{*}$ when $\mathcal{R}_{0}>1$. It can be stated as follows.

### Theorem 2.1

*System* (1.3) *has a disease*-*free equilibrium* $P_{0}$ *as follows*:

$$P_{0}= \biggl(\frac{(\delta_{0}+\eta)A}{\delta_{0}^{2}+(\mu+\eta)\delta _{0}},0,0,0,\frac{A\mu}{\delta_{0}^{2}+(\mu+\eta)\delta_{0}} \biggr), $$

*which exists for all parameter values*. *For* $\mathcal{R}_{0}>1$, *the endemic equilibrium* $P^{*}$ *admits the unique positive equilibrium point for system* (1.3).

### Remark 2.2

From the proof of the existence of endemic equilibrium $P^{*}$, it is not difficult to arrive at such a conclusion that the nonlinear treatment function $g(I)$ has an upper bound $\frac{A\delta_{1}}{m_{2}}$, which is reasonable for limited medical resources in our daily life.

### Proposition 2.1

*The set*

$$\varOmega = \biggl\{ (S,E,I,R,V)\in R_{+}^{5}, 0< S,E,I,R,V,S+E+I+R+V\leq \frac {A}{\delta_{0}} \biggr\} $$

*is a positively invariant and attracting region for the disease transmission model given by system* (1.3) *with initial conditions* (1.4).

### Proof

Summing up the five equations in system (1.3) and denoting

$$ N(t)=S(t)+E(t)+I(t)+R(t)+V(t), $$

we get

$$ \frac{dN(t)}{dt}=A-\delta_{0} N-\delta_{3} I\leq A- \delta_{0}N, $$

i.e.,

$$ \frac{dN(t)}{dt}+\delta_{0} N\leq A. $$

Now integrating both sides of the above inequality and using the theory of differential inequality [41], we obtain

$$ 0< N\leq \biggl(N(0)e^{-\delta_{0}t}+\frac{A}{\delta_{0}}\bigl(1-e^{-\delta _{0}t} \bigr) \biggr). $$

Clearly, $0< N\leq\frac{A}{\delta_{0}}$ as $t\rightarrow\infty$. If $N(0)\leq\frac{A}{\delta_{0}}$, then $N(t)\leq\frac{A}{\delta_{0}}$. Thus, the set *Ω* is positive-invariant, that is, all initial solutions belong to *Ω* remain in *Ω* for all $t>0$. □

## Global stability of the disease-free equilibrium by means of Lyapunov function

In this section, we investigate the global stability of the disease-free equilibrium $P_{0}$ for system (1.3).

### Theorem 3.1

*If* $\mathcal{R}_{0}<1-\frac{Ag'(0)-\delta_{0}g(\frac{A}{\delta _{0}})}{A(m_{3}+g'(0))}$, *then the disease*-*free equilibrium* $P_{0}$ *of system* (1.3) *is globally asymptotically stable in the feasible region* *Ω*. *If* $\mathcal{R}_{0}>1$, *then* $P_{0}$ *is unstable*.

### Proof

The Jacobian matrix of system (1.3) at $P_{0}$ is

$$ J(P_{0})= \begin{pmatrix} -m_{1} & 0 & -\frac{\partial f}{\partial I}(\frac{(\delta_{0}+\eta)A}{\delta _{0}^{2}+(\mu+\eta)\delta_{0}},0) & 0 & \eta \\ 0 & -m_{2} & \frac{\partial f}{\partial I}(\frac{(\delta_{0}+\eta)A}{\delta _{0}^{2}+(\mu+\eta)\delta_{0}},0) & 0 & 0 \\ 0 & \delta_{1} & -m_{3}-g'(0) & 0 & 0 \\ 0 & 0 & \delta_{2}+g'(0) & -\delta_{0} & 0 \\ \mu & 0 & 0 & 0 & -m_{4} \end{pmatrix} . $$

Obviously, $\lambda_{1}=-\delta_{0}$ is an eigenvalue of $J(P_{0})$. The other eigenvalues of $J(P_{0})$ are determined by the equations

$$ \lambda^{2}+(m_{1}+m_{4})\lambda+(m_{1}m_{4}- \mu)=0 $$

and

$$ \lambda^{2}+\bigl(m_{2}+m_{3}+g'(0) \bigr)\lambda+m_{2}\bigl(m_{3}+g'(0)\bigr) (1- \mathcal{R}_{0})=0, $$

respectively. If $\mathcal{R}_{0}>1$, then one eigenvalue is positive. Thus, $P_{0}$ is unstable.

When $\mathcal{R}_{0}<1-\frac{Ag'(0)-\delta_{0}g(\frac{A}{\delta _{0}})}{A(m_{3}+g'(0))}$, to prove the disease-free equilibrium $P_{0}$ is globally asymptotically stable, we consider the Lyapunov function $V(E,I)=\delta_{1}E+m_{2}I$. The derivative of $V(E,I)$ along system (1.3) satisfies

$$\begin{aligned} \frac{dV(E,I)}{dt} =&\delta_{1}\bigl(f(S,I)-m_{2}E \bigr)+m_{2}\bigl(\delta_{1}E-m_{3}I-g(I)\bigr) \\ =&\delta_{1} f(S,I)-m_{2}\bigl(m_{3}I+g(I)\bigr) \\ =&I \biggl(\delta_{1}\frac{f(S,I)}{I}-m_{2} \frac{m_{3}I+g(I)}{I} \biggr) \\ \leq&I \biggl(\delta_{1}\frac{f(\frac{A}{m_{1}-\frac{\mu\eta }{m_{4}}},I)}{I}-m_{2} \biggl(m_{3}+\frac{g(I)}{I}\biggr) \biggr) \\ \leq&I \biggl(\delta_{1}\lim_{I\rightarrow0^{+}} \frac{f(\frac{A}{m_{1}-\frac {\mu\eta}{m_{4}}},I)}{I}-m_{2}\biggl(m_{3}+\frac{g(I)}{I}\biggr) \biggr) \\ =&I \biggl[\delta_{1}\frac{\partial f(\frac{A}{m_{1}-\frac{\mu\eta }{m_{4}}},0)}{\partial I}-m_{2} \biggl(m_{3}+\frac{g(I)}{I}\biggr) \biggr] \\ =&I \biggl[m_{2}\bigl(m_{3}+g'(0)\bigr) \mathcal{R}_{0}-m_{2}\biggl(m_{3}+ \frac{g(I)}{I}\biggr) \biggr] \\ =&I m_{2}\bigl(m_{3}+g'(0)\bigr) \biggl[ \mathcal{R}_{0}-\frac{m_{2}(m_{3}+\frac {g(I)}{I})}{m_{2}(m_{3}+g'(0))} \biggr] \\ =&I m_{2}\bigl(m_{3}+g'(0)\bigr) \biggl[ \mathcal{R}_{0}-1+\frac{m_{3}+g'(0)-m_{3}-\frac {g(I)}{I}}{m_{3}+g'(0)} \biggr] \\ \leq&I m_{2}\bigl(m_{3}+g'(0)\bigr) \biggl[ \mathcal{R}_{0}-1+\frac{Ag'(0)-\delta _{0}g(\frac{A}{\delta_{0}})}{A(m_{3}+g'(0))} \biggr] \\ \leq& 0. \end{aligned}$$

Furthermore, $\frac{dV(E,I)}{dt}=0$ iff $I=0$. Thus, the largest compact invariant set in $\{(S,E,I,R,V)| \dot{V}(E,I)=0\}$, when $\mathcal{R}_{0}<1-\frac{Ag'(0)-\delta_{0}g(\frac{A}{\delta _{0}})}{A(m_{3}+g'(0))}$, is the singleton $P_{0}$. By the LaSalle invariance principle theorem ([42], p. 30), the disease-free equilibrium $P_{0}$ is globally asymptotically stable if $\mathcal{R}_{0}<1-\frac {Ag'(0)-\delta_{0}g(\frac{A}{\delta_{0}})}{A(m_{3}+g'(0))}$. This completes the proof. □

## Global stability of the endemic equilibrium by means of geometric approach

In this section, we analyze the stability of the endemic equilibrium $P^{*}$. First, we show the local stability of the endemic equilibrium of system (1.3) around the endemic equilibrium $P^{*}$.

### Theorem 4.1

*If* $\mathcal{R}_{0}>1$, *then the endemic equilibrium* $P^{*}$ *exists and is locally asymptotically stable if the following conditions hold*:

(i) $\eta<\frac{a_{11}m_{4}}{\mu}$;
(ii) $a_{13}<\min\{\frac{a_{11}m_{4}+(a_{11}+m_{4})(a_{33}+m_{2})+a_{33}m_{2}-\mu\eta }{\delta_{1}}, \frac{a_{11}(a_{33}+m_{2})m_{4}+a_{33}(a_{11}+m_{4})m_{2}-\mu\eta (a_{33}+m_{2})}{(m_{1}+m_{4})\delta_{1}}, \frac{a_{11}a_{33}m_{2}m_{4}-\mu\eta a_{33}m_{2}}{(m_{1}m_{4}-\mu\eta)\delta_{1}}\}$;
(iii) $0< h\leq\frac{z}{a_{33}m_{4}+(a_{33}+m_{4})m_{2}}$ *and* $a_{11}> \frac{(a_{33}\mu m_{2}\eta-a_{21}a_{13}m_{4}\delta_{1})h^{2}-a_{13}\delta _{1}h(\mu\eta h+z)+ a_{33}hz(m_{2}+m_{4})+hz(m_{2}m_{4}-\mu\eta )-z^{2}}{h[a_{33}m_{2}m_{4}h-a_{13}m_{4}\delta_{1}h-(a_{33}+m_{2}+m_{4})z]}$. *Here all the parameters* $a_{11}$, $a_{13}$, $a_{21}$, $a_{33}$, $a_{43}$ *are defined in* (4.1). *The values of* *h* *and* *z* *equal to* $B_{1}$ *and* $B_{3}$, *respectively*.

### Proof

The Jacobian matrix of system (1.3) at $P^{*}$ is given by

$$ J\bigl(P^{*}\bigr)= \begin{pmatrix} -a_{11} & 0 & -a_{13} & 0 & \eta \\ a_{21} & -m_{2} & a_{13} & 0 & 0 \\ 0 & \delta_{1} & -a_{33} & 0 & 0 \\ 0 & 0 & a_{43} & -\delta_{0} & 0 \\ \mu & 0 & 0 & 0 & -m_{4} \end{pmatrix} , $$

where

4.1 $$ \begin{aligned} &a_{11}=\frac{\partial f(S^{*},I^{*})}{\partial S}+m_{1}, \qquad a_{13}=\frac {\partial f(S^{*},I^{*})}{\partial I}, \\ &a_{21}=\frac{\partial f(S^{*},I^{*})}{\partial S},\qquad a_{33}=m_{3}+g' \bigl(I^{*}\bigr),\qquad a_{43}=\delta_{2}+g' \bigl(I^{*}\bigr). \end{aligned} $$

Clearly, one of the roots of $J(P^{*})$ is negative, i.e. $-\delta_{0}$. The remaining roots can be determined from the following equation:

$$ \lambda^{4}+B_{1}\lambda^{3}+B_{2} \lambda^{2}+B_{3}\lambda+B_{4}=0, $$

where

$$\begin{aligned}& B_{1}=a_{11}+a_{33}+m_{2}+m_{4}>0, \\& B_{2}=a_{11}m_{4}+(a_{11}+m_{4}) (a_{33}+m_{2})+a_{33}m_{2}-\mu\eta-\delta _{1}a_{13}, \\& B_{3}=a_{11}(a_{33}+m_{2})m_{4}+a_{33}(a_{11}+m_{4})m_{2}- \mu\eta (a_{33}+m_{2})-\delta_{1}a_{13}(m_{1}+m_{4}), \\& B_{4}=a_{11}a_{33}m_{2}m_{4}-a_{33} \mu\eta m_{2}-\delta_{1}a_{13}m_{1}m_{4}+ \delta _{1}a_{13}\mu\eta. \end{aligned}$$

Using assumptions (i) and (ii), by a direct calculation, we have $B_{i}>0$ for $i=1,2,3,4$. It follows from the Routh–Hurwitz criteria [43] that all the eigenvalues associated to $J(P^{*})$ have negative real parts iff $B_{i}>0$ for $i=1,2,3,4$ and $B_{1}B_{2}B_{3}>B_{3}^{2}+B_{1}^{2}B_{4}$.

Now,

$$\begin{aligned}& B_{1}B_{2}B_{3}-B_{3}^{2}-B_{1}^{2}B_{4} \\& \quad = B_{3}(B_{1}B_{2}-B_{3})-B_{1}^{2}B_{4} \\& \quad = \bigl[a_{11}(a_{33}+m_{2})m_{4}+a_{33}(a_{11}+m_{4})m_{2}- \mu\eta (a_{33}+m_{2})-\delta_{1}a_{13}(m_{1}+m_{4}) \bigr] \\& \qquad {}\cdot \bigl\{ a_{11}(a_{11}+m_{4})m_{4}+(a_{11}+m_{4})^{2}(a_{33}+m_{2})+(a_{11}+m_{4}) (a_{33}+m_{2})^{2} \\& \qquad {}+a_{33}(a_{33}+m_{2})m_{2}-(a_{11}+m_{4}) \mu\eta-\delta _{1}a_{13}(a_{21}+a_{33}+m_{2}) \bigr\} -B_{4}h^{2} \\& \quad = -B_{4}h^{2}+h\bigl[a_{11}m_{4}+(a_{11}+m_{4}) (a_{33}+m_{2})+a_{33}m_{2}-\mu\eta-\delta _{1}a_{13}\bigr]z \\& \qquad {}-\bigl[a_{11}(a_{33}+m_{2})m_{4}+ m_{2}a_{33}(a_{11}+m_{4})-\mu\eta (a_{33}+m_{2}) \\& \qquad {}-\delta_{1}a_{13}(m_{1}+m_{4}) \bigr]z \\& \quad = -B_{4}h^{2}+h\bigl[a_{33}m_{4}+(a_{33}+m_{4})m_{2}+a_{11}(a_{33}+m_{2}+m_{4})- \mu\eta -\delta_{1}a_{13}\bigr]z \\& \qquad {}-\bigl[a_{11}a_{33}m_{4}+a_{33}m_{2} m_{4}+a_{11}(a_{33}+m_{4})m_{2}-(a_{33}+m_{2}) \mu\eta \\& \qquad {}-\delta_{1}a_{13}(m_{1}+m_{4}) \bigr]z \\& \quad = -B_{4}h^{2}+h\bigl[a_{33}m_{4}+(a_{33}+m_{4})m_{2}+a_{11}(a_{33}+m_{2}+m_{4})- \mu\eta -\delta_{1}a_{13}\bigr]z \\& \qquad {}-\bigl[a_{11}a_{33}m_{4}+a_{33}m_{2} m_{4}+a_{11}(a_{33}+m_{4})m_{2}-(a_{33}+m_{2}) \mu\eta \\& \qquad {}-\delta_{1}a_{13}(m_{1}+m_{4}) \bigr]^{2} \\& \quad > 0, \end{aligned}$$

if (iii) holds. This ends the proof. □

To find the global stability of system (1.3), it is necessary to reduce system (1.3) first. Since recovered class *R* does not have any effect on the dynamics of *S*, *V*, *E* and *I* class, we shall investigate the following system:

4.2 $$ \textstyle\begin{cases} \frac{dS}{dt} =A-\delta_{0}S-f(S,I)+\eta V-\mu S, \\ \frac{dE}{dt} =f(S,I)-(\delta_{0}+\delta_{1})E, \\ \frac{dI}{dt} =\delta_{1}E-(\delta_{0}+\delta_{2}+\delta_{3})I-g(I), \\ \frac{dV}{dt} =\mu S-(\delta_{0}+\eta)V. \end{cases} $$

The solutions of (4.2) corresponding to nonnegative initial values remain nonnegative for all time. Moreover, we observe that the total population size of (4.2) denoted by $X(t)$ satisfies $\dot {X}=A-\delta_{0}X-\delta_{2}I-\delta_{3}I-g(I)$, so that we can study the model in the region:

$$\varTheta = \biggl\{ (S,E,I,V)\in R_{+}^{4}:S+E+I+V\leq\frac{A}{\delta_{0}} \biggr\} . $$

Here we follow the approach used in [8] for a SVEIR model of SARS epidemic spread.

Let us consider the following autonomous dynamical system:

4.3 $$ \dot{x}=f(x), $$

where $f:D\rightarrow\mathbf{R}^{n}$, $D\subset\mathbf{R}^{n}$ which is an open set, simply connected and $f\in C^{1}(D)$. Suppose that $x^{*}$ is an equilibrium point of (4.3), i.e. $f(x^{*})=0$. Therefore, $x^{*}$ is said to be globally stable in *D* if it is locally stable and all trajectories in *D* converge to $x^{*}$.

Let $Q(x)$ be a matrix-valued function of order $\bigl ( {\scriptsize\begin{matrix}{} n \cr 2 \end{matrix}} \bigr ) \times \bigl ( {\scriptsize\begin{matrix}{} n \cr 2 \end{matrix}} \bigr ) $ that is $C^{1}$ on *D*. We also consider the matrix *A* which is defined as

$$A=Q_{f}Q^{-1}+QMQ^{-1}, $$

where the matrix $Q_{f}$ is

$$\bigl(q_{ij}(x)\bigr)_{f}=\biggl(\frac{\partial q_{ij}(x)}{\partial x} \biggr)^{T}\cdot f(x)=\nabla q_{ij}\cdot f(x), $$

and the matrix *M* is the second additive compound matrix of the Jacobian matrix *J*. Further the Lozinskiĭ measure *μ̄* of *A* with respect to a vector norm $\|\cdot\|$ can be defined in $\mathbf{R}^{\bigl ( {\scriptsize\begin{matrix}{} n \cr 2 \end{matrix}} \bigr )}$ as follows:

$$\bar{\mu}(A)=\lim_{h\rightarrow0^{+}}\frac{\|I+hA\|}{h}. $$

We will apply the following theorem according to [44].

### Lemma 4.1

*If* $D_{1}$ *is a compact absorbing subset in the interior of* *D*, *and there exist* $\gamma>0$ *and a Lozinskiĭ measure* $\bar{\mu}(A)\leq -\gamma$ *for all* $x\in D_{1}$, *then every omega limit point of system* (4.2) *in the interior of* *D* *is an equilibrium in* $D_{1}$.

Theorem 2.1 states that $\mathcal{R}_{0}>1$ implies the existence and uniqueness of the endemic equilibrium $P^{*}$. Further, we know that the disease-free equilibrium $P_{0}$ is unstable when $\mathcal{R}_{0}>1$. The instability of $P_{0}$, together with $P_{0}\in\partial \varTheta $, which implies the uniform persistence of the state variables (see [45]). Thus, there exists a constant $a>0$ such that any solution $(S(t),E(t),I(t),V(t))$ with $(S(0),E(0),I(0),V(0))$ in the orbit of system (4.2) satisfies

$$\min \Bigl\{ \lim_{t\rightarrow\infty}\inf S(t),\lim_{t\rightarrow\infty } \inf E(t),\lim_{t\rightarrow\infty}\inf I(t),\lim_{t\rightarrow\infty }\inf V(t) \Bigr\} \geq a. $$

The uniform persistence of system (4.2), incorporating the boundedness of *Θ*, suggests that the compact absorbing set in the interior of *Θ*; see [46]. Hence, Lemma 4.1 may be applied, with $D=\varTheta $.

According to [47], the Lozinskiĭ measure in Lemma 4.1 can be evaluated as:

$$\bar{\mu}(A)=\inf\bigl\{ \bar{k}:D_{+}\|\mathbf{z}\|\leq\bar{k}\|\mathbf{z}\| , \text{for all solutions of } \mathbf{z}'=A\mathbf{z}\bigr\} , $$

where $D_{+}$ is the right-hand derivative. The endemic equilibrium is locally asymptotically stable, provided $R_{0}>1$. Hence, to get the global asymptotic stability, according to Lemma 4.1, the trick of the proof is to find a norm $\|\cdot\|$ such that $\bar{\mu}(A)<0$ for all *x* in the interior of *Θ*.

Starting with the Jacobian matrix *J* of (4.2),

$$ J= \begin{pmatrix} a_{11} & a_{12} & a_{13} & a_{14} \\ a_{21} & a_{22} & a_{23} & a_{24} \\ a_{31} & a_{32} & a_{33} & a_{34} \\ a_{41} & a_{42} & a_{43} & a_{44} \end{pmatrix} , $$

the second additive compound matrix is given by

$$ \begin{pmatrix} a_{11}+a_{22} & a_{23} & a_{24} & -a_{13} & -a_{14} & 0 \\ a_{32} & a_{11}+a_{33} & a_{34} & a_{12} & 0 & -a_{14} \\ a_{42} & a_{43} & a_{11}+a_{44} & 0 & a_{12} & a_{13} \\ -a_{31} & a_{21} & 0 & a_{22}+ a_{33} & a_{34} & -a_{24} \\ -a_{41} & 0 & a_{21} & a_{43} & a_{22}+a_{44} & a_{23} \\ 0 & -a_{41} & a_{31} & -a_{42} & a_{32} & a_{33}+a_{44} \end{pmatrix} . $$

Hence, the second additive compound matrix of *J* is given as follows:

$$ M= \begin{pmatrix} M_{11} & M_{12} & 0 & M_{14} & M_{15} & 0 \\ M_{21} & M_{22} & 0 & 0 & 0 & M_{26} \\ 0 & 0 & M_{33} & 0 & 0 & M_{36} \\ 0 & M_{42} & 0 & M_{44} & 0 & 0 \\ M_{51} & 0 & M_{53} & 0 & M_{55} & M_{56} \\ 0 & M_{62} & 0 & 0 & M_{65} & M_{66} \end{pmatrix} , $$

where

$$\begin{aligned}& M_{11}=-2\delta_{0}-\frac{\partial f(S,I)}{\partial S}-(\mu+ \delta_{1}),\qquad M_{12}=\frac{\partial f(S,I)}{\partial I}, \\& M_{14}=\frac{\partial f(S,I)}{\partial I},\qquad M_{15}=-\eta; \\& M_{21}=\delta_{1},\qquad M_{22}=-2 \delta_{0}-\frac{\partial f(S,I)}{\partial S}-\mu-(\delta_{2}+ \delta_{3})-g'(I),\qquad M_{26}=-\eta; \\& M_{33}=-2\delta_{0}-\frac{\partial f(S,I)}{\partial S}-(\mu+\eta),\qquad M_{36}=-\frac{\partial f(S,I)}{\partial I}; \\& M_{42}=\frac{\partial f(S,I)}{\partial S},\qquad M_{44}=-2 \delta_{0}-(\delta _{1}+\delta_{2}+ \delta_{3})-g'(I); \\& M_{51}=-\mu,\qquad M_{53}=\frac{\partial f(S,I)}{\partial S},\qquad M_{55}=-2\delta_{0}-(\delta_{1}+\eta), \\& M_{56}=\frac{\partial f(S,I)}{\partial I},\qquad M_{62}=-\mu,\qquad M_{65}=\delta_{1}; \\& M_{66}=-2\delta_{0}-(\delta_{2}+\delta _{3}+\eta)-g'(I). \end{aligned}$$

Now we consider the following matrix:

4.4 $$ Q= \begin{pmatrix} \frac{1}{I} & 0 & 0 & 0 & 0 & 0 \\ 0 & \frac{1}{I} & 0 & 0 & 0 & 0 \\ 0 & 0 & 0 & \frac{1}{I} & 0 & 0 \\ 0 & 0 & \frac{1}{V} & 0 & 0 & 0 \\ 0 & 0 & 0 & 0 & \frac{1}{V} & 0 \\ 0 & 0 & 0 & 0 & 0 & \frac{1}{V} \end{pmatrix} . $$

Then we obtain the matrix $A=Q_{f}Q^{-1}+QMQ^{-1}$, where $Q_{f}$ is the derivative of *Q* in the direction of the vector field *f*. More accurately, we have

$$ Q_{f}Q^{-1}=-\operatorname{diag} \biggl\{ \frac{\dot{I}}{I},\frac{\dot{I}}{I},\frac{\dot {I}}{I},\frac{\dot{V}}{V}, \frac{\dot{V}}{V},\frac{\dot{V}}{V} \biggr\} . $$

Hence, in view of the fact that

$$\frac{\dot{I}}{I}=\delta_{1}\frac{E}{I}-(\delta_{0}+ \delta_{2}+\delta_{3})-\frac {g(I)}{I},\qquad \frac{\dot{V}}{V}=\mu\frac{S}{V}-(\delta_{0}+\eta), $$

we obtain

$$ A= \begin{pmatrix} A_{11} & A_{12} & A_{13} & 0 & A_{15} & 0 \\ A_{21} & A_{22} & 0 & 0 & 0 & A_{26} \\ 0 & A_{32} & A_{33} & 0 & 0 & 0 \\ 0 & 0 & 0 & A_{44} & 0 & A_{46} \\ A_{51} & 0 & 0 & A_{54} & A_{55} & A_{56} \\ 0 & A_{62} & 0 & 0 & A_{65} & A_{66} \end{pmatrix} , $$

where

$$\begin{aligned}& A_{11}=\frac{g(I)}{I}+\delta_{2}+\delta_{3}- \delta_{0}-\delta_{1}-\delta _{1} \frac{E}{I}-\frac{\partial f(S,I)}{\partial S}-\mu, \\& A_{12}=\frac{\partial f(S,I)}{\partial I},\qquad A_{13}=\frac{\partial f(S,I)}{\partial I}, \qquad A_{15}=-\eta\frac{V}{I}; \\& A_{21}=\delta_{1}, \qquad A_{22}=- \delta_{0}-\frac{\partial f(S,I)}{\partial S}-\mu-g'(I)+ \frac{g(I)}{I}-\delta_{1}\frac{E}{I},\qquad A_{26}=-\eta\frac {V}{I}; \\& A_{32}=\frac{\partial f(S,I)}{\partial S},\qquad A_{33}= \frac {g(I)}{I}-(\delta_{0}+\delta_{1})-g'(I)- \delta_{1}\frac{E}{I}; \\& A_{44}=-\delta_{0}-\frac{\partial f(S,I)}{\partial S}-\mu-\mu \frac {S}{V},\qquad A_{46}=-\frac{\partial f(S,I)}{\partial I}; \\& A_{51}=-\mu\frac{I}{V},\qquad A_{54}= \frac{\partial f(S,I)}{\partial S},\qquad A_{55}=-\mu\frac{S}{V}-( \delta_{0}+\delta_{1}),\qquad A_{56}= \frac{\partial f(S,I)}{\partial I}; \\& A_{62}=-\mu\frac{I}{V},\qquad A_{65}= \delta_{1},\qquad A_{66}=-(\delta_{0}+\delta _{2}+\delta_{3})-g'(I)-\mu\frac{S}{V}. \end{aligned}$$

As in [8], we consider the following norm on $R^{6}$:

4.5 $$ \Vert \mathbf{z} \Vert =\max\{U_{1},U_{2} \}, $$

where $\mathbf{z}\in\mathbf{R}^{6}$, with components $z_{i}$, $i=1,2,\ldots ,6$, and $U_{1}(z_{1},z_{2},z_{3})$ is defined as:

$$\begin{aligned}& U_{1}(z_{1},z_{2},z_{3}) \\& \quad = \textstyle\begin{cases} \max\{|z_{1}|,|z_{2}|+|z_{3}|\},& \text{if } \operatorname {sgn}(z_{1})=\operatorname{sgn}(z_{2})=\operatorname{sgn}(z_{3}), \\ \max\{|z_{2}|,|z_{1}|+|z_{3}|\},& \text{if } \operatorname {sgn}(z_{1})=\operatorname{sgn}(z_{2})=-\operatorname{sgn}(z_{3}), \\ \max\{|z_{1}|,|z_{2}|,|z_{3}|\},& \text{if } \operatorname {sgn}(z_{1})=-\operatorname{sgn}(z_{2})=\operatorname{sgn}(z_{3}), \\ \max\{|z_{1}|+|z_{3}|,|z_{2}|+|z_{3}|\},& \text{if } -\operatorname {sgn}(z_{1})=\operatorname{sgn}(z_{2})=\operatorname{sgn}(z_{3}), \end{cases}\displaystyle \end{aligned}$$

and $U_{2}(z_{4},z_{5},z_{6})$ is defined as

$$\begin{aligned}& U_{2}(z_{4},z_{5},z_{6}) \\& \quad = \textstyle\begin{cases} |z_{4}|+|z_{5}|+|z_{6}| & \text{if } \operatorname{sgn}(z_{4})=\operatorname {sgn}(z_{5})=\operatorname{sgn}(z_{6}), \\ \max\{|z_{4}|+|z_{5}|,|z_{4}|+|z_{6}|\},& \text{if } \operatorname {sgn}(z_{4})=\operatorname{sgn}(z_{5})=-\operatorname{sgn}(z_{6}), \\ \max\{|z_{5}|,|z_{4}|+|z_{6}|\},& \text{if } \operatorname {sgn}(z_{4})=-\operatorname{sgn}(z_{5})=\operatorname{sgn}(z_{6}), \\ \max\{|z_{4}|+|z_{6}|,|z_{5}|+|z_{6}|\},& \text{if } -\operatorname {sgn}(z_{4})=\operatorname{sgn}(z_{5})=\operatorname{sgn}(z_{6}). \end{cases}\displaystyle \end{aligned}$$

In the next, we will use the following inequalities:

$$|z_{1}|,|z_{2}|,|z_{3}|,|z_{2}+z_{3}| \leq U_{1} $$

and

$$|z_{i}|,|z_{i}+z_{j}|,|z_{4}+z_{5}+z_{6}| \leq U_{2}(z),\quad i=4,5,6; i\neq j. $$

Furthermore, we assume that

4.6 $$ \delta_{2}+\delta_{3}>\delta_{1}. $$

We will use the inequalities mentioned above to get the estimates on $D_{+}\|\mathbf{z}\|$.

### Theorem 4.2

*For* $\mathcal{R}_{0}>1$, *system* (4.2) *admits an unique endemic equilibrium which is globally asymptotically stable in the interior of* *Θ*, *provided that inequality* (4.6) *is satisfied and that*

4.7 $$ \max \{\delta_{2}+\delta_{3}+\blacktriangle, \delta_{1}-\delta _{0}+\blacktriangledown \}< -\omega $$

*for some positive constant* *ω*, *where*

$$\begin{aligned}& \blacktriangle=\sup_{t\in(0,\infty)}\frac{g(I)}{I}-\sup _{t\in(0,\infty )}\frac{\delta_{1}E}{I}+\sup_{t\in(0,\infty)} \frac{\eta V}{I} +\sup_{t\in(0,\infty)} \biggl\{ \frac{\partial f(S,I)}{\partial S}, \frac {\partial f(S,I)}{\partial I} \biggr\} , \\& \blacktriangledown=\sup_{t\in(0,\infty)}\frac{2\mu I}{V}-\sup _{t\in (0,\infty)}\frac{\mu S}{V}+\sup_{t\in(0,\infty)} \frac{\partial f(S,I)}{\partial I}. \end{aligned}$$

### Proof

The basic idea of the proof is to obtain the estimate of the right derivate $D_{+}\|\mathbf{z}\|$ of the norm (4.5). For this purpose, we need to discuss sixteen cases according to the different orthants and the definition of the norm (4.5) within each orthant.

*Case* 1:

4.8 $$ U_{1}>U_{2} \text{ and } z_{1},z_{2},z_{3}>0 \text{ with } |z_{1}|>|z_{2}|+|z_{3}|. \text{ Then}, \|\mathbf{z}\|=|z_{1}|. $$

This shows that

$$\begin{aligned} D_{+}\|\mathbf{z}\| =&z'_{1} \\ =&A_{11}z_{1}+A_{12}z_{2}+A_{13}z_{3}+A_{15}z_{5} \\ \leq& \biggl[\frac{g(I)}{I}+\delta_{2}+\delta_{3}- \delta_{0}-\delta_{1}-\delta _{1} \frac{E}{I}-\frac{\partial f(S,I)}{\partial S}-\mu \biggr]|z_{1}| \\ &{}+ \frac{\partial f(S,I)}{\partial I}\bigl( \vert z_{2} \vert +|z_{3}|\bigr)+\eta\frac{V}{I}|Z_{5}|. \end{aligned}$$

By using $|z_{5}|< U_{2}<|z_{1}|$ and $|z_{2}|+|z_{3}|<|z_{1}|$, it follows from (4.8) that

$$\begin{aligned}& D_{+}\|\mathbf{z}\| \\& \quad \leq \biggl[\frac{g(I)}{I}+\delta_{2}+\delta_{3}- \delta_{0}-\delta_{1}-\delta _{1} \frac{E}{I}-\frac{\partial f(S,I)}{\partial S}-\mu+\frac{\partial f(S,I)}{\partial I}+\eta \frac{V}{I} \biggr]\|\mathbf{z}\|. \end{aligned}$$

*Case* 2:

4.9 $$ U_{1}>U_{2} \text{ and } z_{1},z_{2},z_{3}>0 \text{ with } |z_{1}|< |z_{2}|+|z_{3}|. \text{ Then}, \|\mathbf{z}\|=|z_{2}|+|z_{3}|. $$

Thus, we have

$$\begin{aligned} D_{+}\|\mathbf{z}\| =&z'_{2}+z'_{3} \\ =&A_{21}z_{1}+A_{22}z_{2}+A_{26}z_{6}+A_{32}z_{2}+A_{33}z_{3} \\ \leq&\delta_{1}|z_{1}|+ \biggl(\frac{g(I)}{I}-g'(I)- \delta_{1}\frac{E}{I} \biggr) \bigl( \vert z_{2} \vert +|z_{3}|\bigr)+\eta\frac{V}{I}|z_{6}|. \end{aligned}$$

Using $|z_{6}|< U_{2}<|z_{2}|+|z_{3}|$ and $|z_{1}|<|z_{2}|+|z_{3}|$, in view of (4.9), one has

$$ D_{+}\|\mathbf{z}\|\leq \biggl[\delta_{1}+\frac{g(I)}{I}-g'(I)- \delta_{1}\frac {E}{I}+\eta\frac{V}{I} \biggr]\| \mathbf{z}\|. $$

The discussion for the other fourteen cases are similar to the ones discussed in [7] and so we omit it here. Thus, we can get the following estimate:

$$D_{+}\|\mathbf{z}\|\leq\max \{\delta_{2}+\delta_{3}+ \blacktriangle,\delta _{1}-\delta_{0}+\blacktriangledown \}\| \mathbf{z}\|, $$

where

$$\begin{aligned}& \blacktriangle=\sup_{t\in(0,\infty)}\frac{g(I)}{I}-\sup _{t\in(0,\infty )}\frac{\delta_{1}E}{I}+\sup_{t\in(0,\infty)} \frac{\eta V}{I} +\sup_{t\in(0,\infty)} \biggl\{ \frac{\partial f(S,I)}{\partial S}, \frac {\partial f(S,I)}{\partial I} \biggr\} , \\& \blacktriangledown=\sup_{t\in(0,\infty)}\frac{2\mu I}{V}-\sup _{t\in (0,\infty)}\frac{\mu S}{V}+\sup_{t\in(0,\infty)} \frac{\partial f(S,I)}{\partial I}. \end{aligned}$$

Now the global stability follows from Lemma 4.1. □

### Remark 4.1

As pointed out by Buonomo and Lacitignola [7], in some real situations, different choices of the matrix *Q* and of the vector norm $\|\cdot\|$ may lead to better sufficient conditions than those we presented here, in the sense that the assumptions on the parameters may be weakened. Thus, it is worth to note that sufficient conditions (4.6) and (4.7) in Theorem 4.2 are derived from the application of the method and numerical simulations suggest that they may be not necessary (see Example 5.1).

## Numerical simulations

The aim of this section is to give a numerical example to illustrate our main results.

### Example 5.1

Consider the system

5.1 $$ \textstyle\begin{cases} \frac{dS}{dt} =A-\delta_{0} S-\frac{mSI}{1+nI}+\eta V-\mu S, \\ \frac{dE}{dt} =\frac{mSI}{1+nI}-(\delta_{0}+\delta_{1})E, \\ \frac{dI}{dt} =\delta_{1}E-(\delta_{0}+\delta_{2}+\delta_{3})I-\frac{\gamma I}{I+a}, \\ \frac{dR}{dt} =\delta_{2}I-\delta_{0}R+\frac{\gamma I}{I+a}, \\ \frac{dV}{dt} =\mu S-(\delta_{0}+\eta)V, \end{cases} $$

which is a particular case of system (1.3) by letting $f(S,I)=\frac{mSI}{1+nI}$ and $g(I)=\frac{\gamma I}{I+a}$, where *m*, *n*, *γ*, *a* are positive and $na>1$. The other parameters in (5.1) have the same biological meanings as in model (1.3).

**Figure 1:**
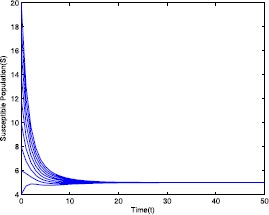
Time series plot of the susceptible population for $\mathcal {R}_{0}=0.571429<0.910714$ with various initial conditions, parameter values are given in Table 1

**Figure 2:**
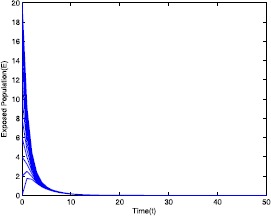
Time series plot of the exposed population for $\mathcal {R}_{0}=0.571429<0.910714$ with various initial conditions, parameter values are given in Table 1

**Figure 3:**
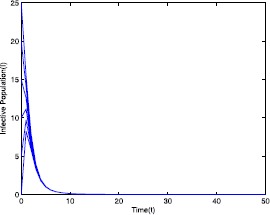
Time series plot of the infective population for $\mathcal {R}_{0}=0.571429<0.910714$ with various initial conditions, parameter values are given in Table 1

**Figure 4:**
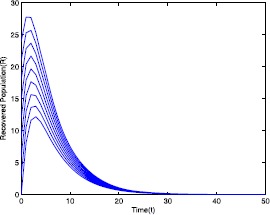
Time series plot of the recovered population for $\mathcal {R}_{0}=0.571429<0.910714$ with various initial conditions, parameter values are given in Table 1

**Figure 5:**
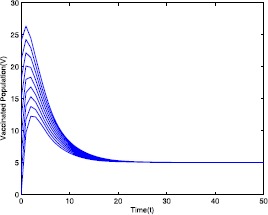
Time series plot of the vaccinated population for $\mathcal {R}_{0}=0.571429<0.910714$ with various initial conditions, parameter values are given in Table 1

We first consider the case when

$$\mathcal{R}_{0}=0.571429< 1-\frac{Ag'(0)-\delta_{0}g(\frac{A}{\delta _{0}})}{A(m_{3}+g'(0))}=0.910714 $$

by using the parameter values given in Table 1. Using these parameter values, for different initial conditions the dynamics of model (5.1) is presented in Figs. 1–5. It shows that system (5.1) has a disease-free equilibrium and it is globally asymptotically stable. This numerical verification supports the result stated in Theorem 3.1.

**Table 1:** Parameter for Figs. 1–5

Parameter	Values
*A*	2
$\delta_{0}$	0.2
*m*	0.2
*n*	2
*η*	0.2
*μ*	0.4
$\delta_{1}$	0.8
$\delta_{2}$	0.5
$\delta_{3}$	0.55
*γ*	0.3
*a*	2

**Figure 6:**
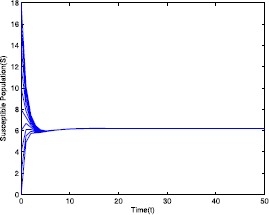
Time series plot of the susceptible population for $\mathcal {R}_{0}=2.211436>1$ with various initial conditions, parameter values are given in Table 2

**Figure 7:**
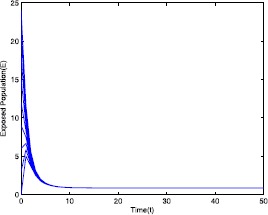
Time series plot of the exposed population for $\mathcal {R}_{0}=2.211436>1$ with various initial conditions, parameter values are given in Table 2

**Figure 8:**
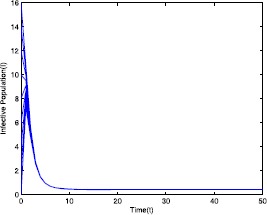
Time series plot of the infective population for $\mathcal {R}_{0}=2.211436>1$ with various initial conditions, parameter values are given in Table 2

**Figure 9:**
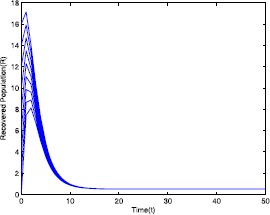
Time series plot of the recovered population for $\mathcal {R}_{0}=2.211436>1$ with various initial conditions, parameter values are given in Table 2

**Figure 10:**
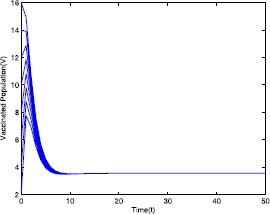
Time series plot of the vaccinated population for $\mathcal {R}_{0}=2.211436>1$ with various initial conditions, parameter values are given in Table 2

Next, we consider the case when $\mathcal{R}_{0}=2.211436>1$ using the parameter values given in Table 2. Using these parameter values, for different initial conditions the dynamics of model (5.1) is presented in Figs. 6–10. It shows that system (5.1) has an endemic equilibrium and it is globally asymptotically stable with different initial values, which supports our analytical results stated in Theorem 4.2.

**Table 2:** Parameter for Figs. 6–10

Parameter	Values
*A*	6
$\delta_{0}$	0.5
*m*	0.8
*n*	2
*η*	0.2
*μ*	0.4
$\delta_{1}$	0.8
$\delta_{2}$	0.5
$\delta_{3}$	0.55
*γ*	0.3
*a*	2

## Conclusions

In this paper, we have considered an SVEIR epidemic model with general nonlinear incidence rate. In model (1.3), we have divided the total population into five compartments, namely susceptible, exposed, infective, recovered, vaccinated population and investigated the dynamical behavior of this model. Here, we have found that

$$\mathcal{R}_{0}=\frac{\delta_{1}}{m_{2}(m_{3}+g'(0))}\frac{\partial f}{\partial I} \biggl( \frac{A}{m_{1}-\frac{\mu\eta}{m_{4}}},0 \biggr) $$

is a basic reproduction number of system (1.3), which helps us to determine the dynamical behavior of the system. We have showed that system (1.3) to be globally asymptotically stable at disease-free equilibrium $P_{0}$ when

$$\mathcal{R}_{0}< 1-\frac{Ag'(0)-\delta_{0}g(\frac{A}{\delta_{0}})}{A(m_{3}+g'(0))}. $$

When $\mathcal{R}_{0}>1$, the endemic equilibrium stable both locally and globally has been derived and analyzed under some conditions. The important mathematical findings for the dynamical behavior of model (1.3) have also numerically been verified for a special case of model (1.3). We would like to point out that the model considered in this paper is not a case study and so it is difficult to choose parameter values from quantitative estimation. We have used hypothetical sets of parameters to verify our analytical results. It is worth to mention that the results presented in this paper improve and extend some related results in [9, 10, 12, 13].

Finally, we remark that there are quite a few spaces to deserve further investigation. For example, we can continue the research in this line considering the vaccination rate *μ* in our model (1.3) as a continuous function, and, later, a discontinuous function. On the other hand, as is well known, epidemiological models which incorporate the control strategies can be useful to both control the spread of disease and minimize the intervention costs. For our model, it is natural to consider vaccination rate coefficient as a control to reduce the disease burden. Thus, it is important and interesting to prove the existence of optimal control, characterize the optimal control, prove the uniqueness of optimal control, compute the optimal control numerically and investigate how the optimal control depends on various parameters in the models. We will devote to these questions our future work.

## Abbreviations

(SVEIR): Susceptible–Vaccinated–Exposed–Infectious–Recovered
